# Proteasome-Rich PaCS as an Oncofetal UPS Structure Handling Cytosolic Polyubiquitinated Proteins. In Vivo Occurrence, in Vitro Induction, and Biological Role

**DOI:** 10.3390/ijms19092767

**Published:** 2018-09-14

**Authors:** Enrico Solcia, Vittorio Necchi, Patrizia Sommi, Vittorio Ricci

**Affiliations:** 1 Department of Molecular Medicine, University of Pavia, 27100 Pavia, Italy; vittorio.necchi@unipv.it (V.N.); patrizia.sommi@unipv.it (P.S.); vricci@unipv.it (V.R.); 2 Pathologic Anatomy Unit, Fondazione IRCCS Policlinico San Matteo, 27100 Pavia, Italy; 3 Centro Grandi Strumenti, University of Pavia, 27100 Pavia, Italy

**Keywords:** proteasome, polyubiquitinated proteins, heat-shock proteins, PaCS, neoplastic cells, fetal cells, microbial oncogenic proteins, trophic factors/interleukins, sequestosomes, aggresomes

## Abstract

In this article, we outline and discuss available information on the cellular site and mechanism of proteasome interaction with cytosolic polyubiquitinated proteins and heat-shock molecules. The particulate cytoplasmic structure (PaCS) formed by barrel-like particles, closely reproducing in vivo the high-resolution structure of 26S proteasome as isolated in vitro, has been detected in a variety of fetal and neoplastic cells, from living tissue or cultured cell lines. Specific trophic factors and interleukins were found to induce PaCS during in vitro differentiation of dendritic, natural killer (NK), or megakaryoblastic cells, apparently through activation of the MAPK-ERK pathway. Direct interaction of CagA bacterial oncoprotein with proteasome was shown inside the PaCSs of a *Helicobacter pylori*-infected gastric epithelium, a finding suggesting a role for PaCS in CagA-mediated gastric carcinogenesis. PaCS dissolution and autophagy were seen after withdrawal of inducing factors. PaCS-filled cell blebs and ectosomes were found in some cells and may represent a potential intercellular discharge and transport system of polyubiquitinated antigenic proteins. PaCS differs substantially from the inclusion bodies, sequestosomes, and aggresomes reported in proteinopathies like Huntington or Parkinson diseases, which usually lack PaCS. The latter seems more linked to conditions of increased cell proliferation/differentiation, implying an increased functional demand to the ubiquitin–proteasome system.

## 1. Introduction

Proteasomes, polyubiquitinated proteins (pUbPs), and heat-shock proteins are highly interconnected functionally in the ubiquitin–proteasome system (UPS), a master regulator of cellular-protein renewal. A UPS-mediated, endoplasmic reticulum (ER)-associated degradative (ERAD) quality-control system has been identified that takes care of secretory proteins accumulating inside ER cisternae [1,2]. Part of the cytoplasmic proteasome has been found, with cytochemical and biochemical investigations, to be associated with ER [3,4]. In addition, a proteasome associated with ER or mixed endosomal-ER or phagosomal-ER compartments, with special reference to IFN-gamma-inducible immunoproteasome, is also likely to have a role in microbial-antigen processing and cross-presentation after coupling with MHC molecules inside ER cisternae [5,6,7,8,9]. However, the precise site of UPS-component interaction to implement quality-control mechanisms of autologous, cytosolic proteins, unrelated to ER, secretion, or cross-presentation, remains less clearly defined [1,2].

It is generally agreed that chaperon molecules like heat-shock protein (Hsp) 70 and Hsp40 interact with newly synthesized proteins while they are just being sorted out from polyribosomes, thus helping them to reach appropriate folding [10]. Hsp70, Hsp90, and their cofactors take care of uncorrected, irreversibly misfolded proteins and selectively promote their polyubiquitination and proteasome-mediated degradation [11,12,13]. In normal cells, most proteasome molecules are dispersed inside ribosome-rich cytoplasm, where they continuously diffuse and freely interact with their functional partners [14], including ribosome-linked factors like translation elongation factors, which may have a direct role in cotranslationally degrading misfolded proteins [15]. Thus, ribosome-rich cytoplasm should be the most likely site of any quality-control mechanism for endogenous cytosolic proteins.

Proteasomes degrade pUbPs to oligopeptides, of which very few molecules (about 1 in 1000 or fewer [16]) reach the transporter for antigen processing (TAP), enter the ER, and couple with MHC molecules, to be finally presented at the cell membrane of antigen-presenting cells (APCs). Therefore, UPS-mediated protein degradation, which is enormously in excess of the few antigenic peptides required by its immune function, largely serves to the cell to remove, by a carefully controlled selective process, a mass of misfolded, translationally or post-translationally defective or denatured proteins, while providing the amino acids necessary for their resynthesis [16]. When anything goes wrong in the process, or this is insufficient in respect to an excessively increased mass of altered proteins, protein-inclusion bodies, sequestosomes, aggresomes, and/or autophagolysosomes may form inside cells.

## 2. Particulate Cytoplasmic Structure (PaCS), an Oncofetal Cytoplasmic Structure Concentrating Proteasome Particles, PUbPs, and Heat-Shock Proteins

**Figure 1:**
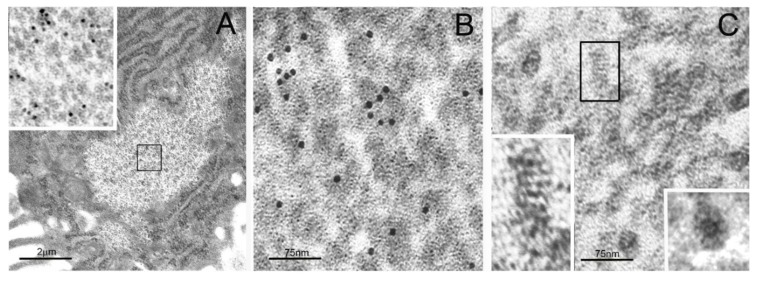
Transmission electron microscopy (TEM) of particulate cytoplasmic structure (PaCS) and high-resolution structure of its proteasome-reactive barrel-like particles. (**A**) Foveolar cell of *Helicobacter pylori*-infected human gastric epithelium showing a clear cytoplasmic area filled with particles, enlarged in the inset (from the boxed area in (**A**); 60,000×), to recognize their barrel-like structure and 20S proteasome immunogold reactivity, typical of PaCS. Note ribosome-rich endoplasmic reticulum (ER) surrounding the PaCS. (**B**) Enlarged PaCS particles showing 19S proteasome immunoreactivity, while in (**C**) a longitudinally oriented particle is boxed and further enlarged in the left-bottom inset (600,000×) to show a side view of the four 20S-core parallel rings, apparently capped at both higher and lower extremities with a 19S regulatory component. Compare with Figures 1, 3, and 4 of Reference [20]. In the bottom right inset of (**C**), a top view of another particle exhibiting the known seven-fold starlike symmetry of the proteasome particle. Reproduced and adapted from Reference [17], under a Creative Commons Attribution (CC BY 4.0) International License.

We recently detected, by extensive ultrastructural and cytochemical analysis of neoplastic, chronically infected, mutated, or fetal cells and tissues, a PaCS (Figure 1A) mainly characterized by a collection of proteasome-immunoreactive barrel-like particles (around 13 nm thick × 15–30 nm or more long, also depending on their orientation in respect to the section plan) [17,18]. At high-resolution electron microscopy (Figure 1B,C), such particles were highly reminiscent of proteasome machinery particles as isolated in vitro and analyzed ultrastructurally by Baumeister and colleagues [19,20].

**Figure 2:**
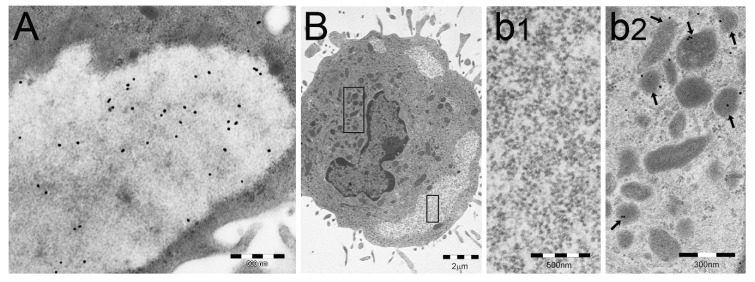
PaCSs store polyubiquitinated proteins (pUbPs) of non-K63-linked type. (**A**,**B**) Human dendritic cells (DCs) differentiated in vitro through GM-CSF plus IL-4 treatment, followed by LPS-induced maturation. In (**A**), PaCS shows immunoreactivity for the FK1 antibody, directed against pUbPs. In (**B**), another human DC from the same preparation shows three PaCSs, one of which (boxed) is enlarged in (**b1**) to illustrate its unreactivity for the anti-K63-linked pUbPs antibody, which, however, reacts with some juxtanuclear late endosomal bodies (left box in (**B**), enlarged in (**b2**): see arrows). (**B**) is reproduced and adapted from Reference [26], under Creative Commons Attribution (CC BY 4.0) International Licenses.

The PaCS, which is usually surrounded by ribosome-rich cytoplasm, with or without rough ER, is easily recognized under transmission electron microscopy (TEM) by its distinctive ultrastructure and its proteasome immunoreactivity with both 20S- and 19S-directed antibodies. It may also be detected under confocal microscopy by proteasome immunofluorescence of tissue sections or glass-adhering cells, provided that they are fixed in formaldehyde-glutaraldehyde/osmium tetroxide solutions [17,21,22]. In addition to proteasome particles, the PaCS also shows selective immunoreactivity for several Hsps [23,24] and for the pUbP-specific FK1 antibody [25] that, coupled with unreactivity for antibodies directed against K63-linked pUbPs, suggests K48-linked pUbPs as likely partners of the PaCS proteasome (Figure 2). Indeed, PaCS′ simultaneous concentration of Hsp70 and Hsp90, with their established role in misfolded/denatured-protein recognition and triage [12,13], together with K48-linked pUbPs [26,27] and proteasome particles, points to PaCS, an essentially cytosolic structure, as a UPS center handling cytosolic proteins. This conclusion is further supported by the detection inside PaCSs of chymotrypsin-type activity (one of the three specific proteasomal enzymes) against fluorogenic model peptides [21].

Given the usually prompt degradation of pUbPs when incubated in vitro with proteasome, the simultaneous accumulation of both UPS components inside PaCSs might seem surprising. However, it should be recalled that several additional molecules are known to interact with UPS inside the cells, among which ubiquitin-activating enzyme E1 [28], E2 and E3 ligases [11], and deubiquitinases [29]. E1 and, especially, Hsps have been found to be highly concentrated inside PaCSs [17,23,24]. Hsp90 seems relevant in this respect as it has been shown to bind and stabilize a large number of so-called “client proteins” forming multiple complexes where they escape degradation [30]. In fact, there is compelling evidence of simultaneously increased intracellular proteasome, misfolded pUbPs, Hsps, and various ubiquitin-related factors, especially inside neoplastic cells [28,30,31,32,33].

## 3. Distribution of PaCS in Fetal and Neoplastic Cells

### 3.1. Fetal Tissues

We found ultrastructurally and cytochemically characteristic PaCSs in the human intestine from 12 to 20-week fetuses in epithelial cells undergoing differentiation towards absorptive enterocytes or enteroendocrine cells [22]. In addition, PaCSs were detected in differentiating rynopharingeal epithelium and in condensing chondroblasts of pharyngeal pouches from E14 to 15.5-day mouse embryos. No PaCSs were observed in fibroblasts or angiopoietic cells from the same tissue preparations and in specimens taken from corresponding normal adult intestine or pharynx.

Given the crucial role played by the UPS in fetal development [34,35], our findings were not surprising. Considering the crucial activity reported, in fetuses, of several trophic factors, including EGF, its receptors, and components of the MAPK-ERK pathway [36,37], a potential role of trophic factors in the process leading to PaCS genesis seemed worth consideration and investigation.

### 3.2. In Neoplastic and Preneoplastic Cells

Among neoplasms with PaCS-positive cells were a number of adenocarcinomas from the kidneys, ovaries, thyroid, gastrointestinal tract, pancreas, liver, and lungs, with special reference to clear-cell, glycogen-rich neoplasms and irrespective of their histologic grade [18]. Among hematologic neoplasms, prominent PaCSs with distintive proteasome particles, FK1 antibody-reactive pUbPs, and Hsp 70 and 90 were found in chronic myeloid leukemia and, less frequently, in myelodysplastic syndromes or myelofibrosis [24]. Surprisingly, no PaCSs were detected in multiple myeloma, despite the known excessive UPS expression in this neoplasm and its sensitivity to proteasome-inhibitor therapy [28,38]. Our investigation of myeloma cells showed, however, a selective increase of ER-associated proteasome [24], suggesting a selective involvement of this proteasome subset in multiple myeloma cells, in accordance with the secretory nature of the immunoglobulins they produce and the ER stress they develop when treated with proteasome inhibitors [39].

In both epithelial and hematologic PaCS-positive neoplasms, proteasome, pUbPs, and Hsps were found to be largely overexpressed, either cytochemically in TEM sections or by immunoblotting of cell lysates [18,24], thus confirming previous findings on tumor extracts or serum of tumor-bearing patients [32,40] and linking them, at least in part, to PaCS itself.

**Figure 3:**
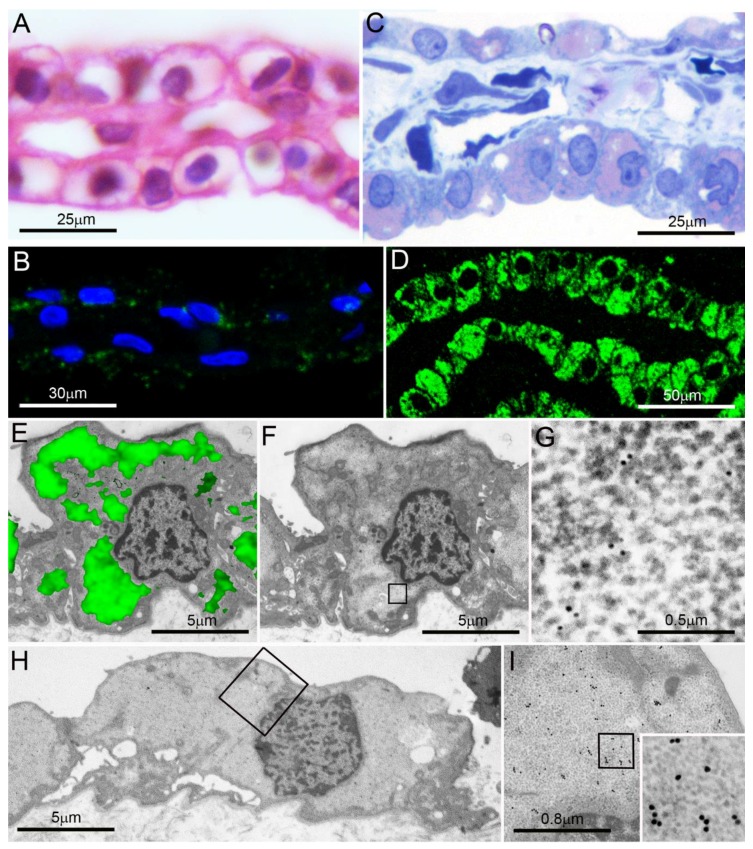
Pancreatic serous microcystic neoplasm. (**A**,**B**) Formalin-fixed paraffin sections; note the clear, apparently “empty” cytoplasm of most cells in (**A**) (hematoxylin-eosin) and their poor reactivity in (**B**) to proteasome immunofluorescence under confocal microscopy (blue: nuclei; green: proteasome). (**C**,**D**) Semithin, aldehyde–osmium-fixed resin sections from the same tumor as in (**A**) and (**B**) show abundant cytoplasmic PaCSs metachromatically stained pink with toluidine blue (**C**) and extensively proteasome immunofluorescent under confocal microscopy ((**D**), green: proteasome). (**E**) Proteasome immunofluorescence (green) of a cell from the same tumor as in (**A**–**D**) is overlapped over its TEM micrograph (alone in (**F**)): note the correspondence of immunofluorescent areas (in (**E**)) with clear PaCS areas (in (**F**)), a finding in keeping with the 20S proteasome immunoreactive particles found in (**G**) (enlarged from the boxed clear area in (**F**)). In (**H**), enlarged in (**I**) and further in its inset (50,000×), PaCS 19S immunoreactive particles fill most cytoplasm in a cell from the same tumor. Reproduced and adapted from Reference [18] under a Creative Commons Attribution (CC BY 4.0) International License.

Of special interest was the pancreatic serous cystic neoplasm (PSCN), which showed abundant PaCSs filling a large part of its clear-cell cytoplasm (Figure 3) [18]. Notably, this tumor has been found to express markedly increased (more than fifty times the normal pancreas values) amounts of the EGF receptor (EGFR), its phosphorylated species, as well as its target MAPK and phosphorylated MAPK, thus suggesting a massive hyperfunction of the EGF-activated signaling pathway [41]. No mutations were found in the *EGFR* gene or in other functionally related genes, such as *KRAS*, *BRAF*, or *PIK3CA*, and the cause of this hyperfunction, coupled with an increased copy number of *EGFR* transcripts in the absence of gene amplification, remained unknown.

In this context, it may be worth recalling that PSCNs, both sporadic and in association with Von Hippel–Lindau (VHL) disease, have been shown: (a) to constitutively express nuclear hypoxia-inducible factor (HIF-1alpha) [42], a transcriptional factor known to activate many neoplasia-associated target genes related with cell survival, proliferation, angiogenesis, and metabolism, and (b) to frequently display mutations of the *VHL* gene [43,44,45], which codes for the E3 ubiquitin ligase-promoting UPS-dependent HIF-1alpha degradation. Thus, it seems possible that impaired VHL function causes HIF-1alpha stabilization, leading to UPS stress and PaCS development. This PSCN-promoting mechanism would be substantially akin to the one involved in the genesis of VHL disease itself and the array of associated neoplasms, including, besides PSCN, clear-cell kidney cancer [46], where plenty of PaCSs have been also detected, even in sporadic neoplasms [22].

**Figure 4:**
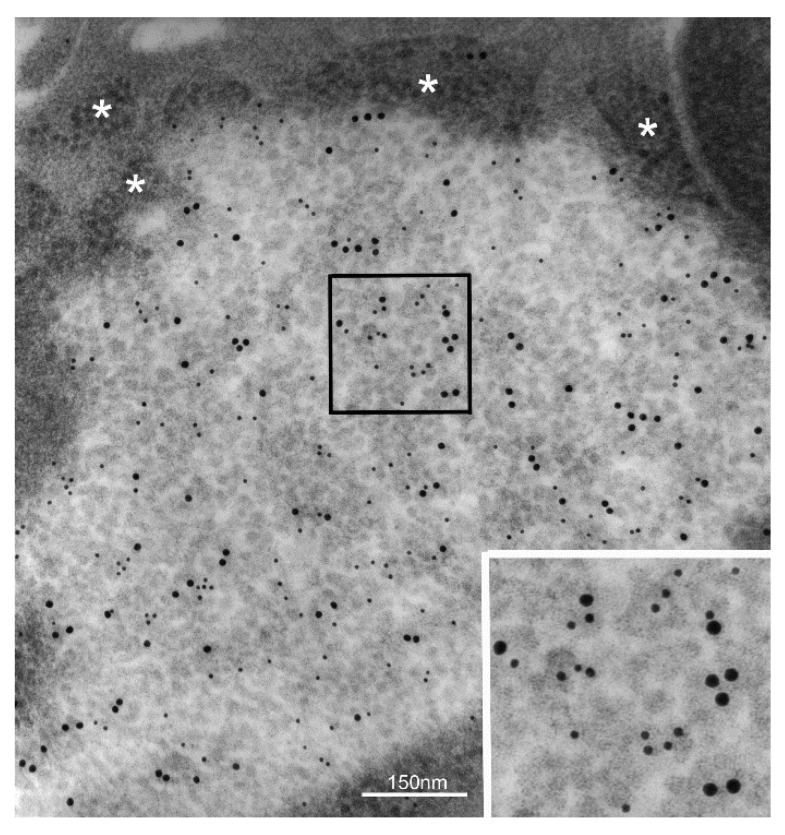
*H. pylori* oncogenic protein CagA concentrates inside PaCS. PaCS from an *H. pylori*-infected human gastric epithelium (enlarged in the inset; 100,000×) shows selective immunogold reactivity for the bacterial oncogenic protein CagA (small gold particles) in addition to 19S proteasome (large gold particles). Note ribosomes (asterisks) in the cytoplasm surrounding PaCS.

PSCN findings again call attention to a possible role of trophic factors in the genesis of PaCSs in neoplastic cells and related preneoplastic conditions. Gastric epithelial carcinogenesis offers a useful paradigm in this respect as we detected PaCSs in all its steps, from *H. pylori*-induced chronic gastritis to dysplastic lesions and full-blown cancer [17,18,47]. Indeed, the *H. pylori* oncoprotein CagA, well known to have a crucial role in most aspects of gastric carcinogenesis, including activation of the RAS-MAPK-ERK pathway [48,49], has been shown to transactivate the EGFR in gastric epithelial cells, thus outlining a potential carcinogenetic role of EGF trophic factor [50]. We investigated the fate of CagA inside *H. pylori*-infected human gastric epithelium using specific anti-CagA antibodies, and found a selective concentration of CagA immunoreactivity inside PaCS (Figure 4). The potential relevance of this finding stems from the fact that CagA itself has been shown to interact with the UPS to induce proteasome-mediated degradation of oncosuppressor proteins like p53 or RUNX3 [51,52,53], thus linking CagA directly with UPS. Indeed, PaCSs may represent a preferential site of the CagA–UPS interaction promoting gastric-cancer development.

Among PaCS-positive neoplastic cell lines, HeLa cells are special in that they take origin from cervical cancer infected with an oncogenic small-DNA virus (i.e., HPV) integrated in the host genome, which in culture persistently expresses the two viral oncoproteins E6 and E7, essential for tumor-cell replication [54,55]. Interestingly, the E6 oncoprotein has been found to promote p53 protein polyubiquitination and proteasomal degradation, thus depriving the cell of its oncosuppressor (proapoptotic) activity [56]. Of course, this HeLa cell tumorigenic mechanism of E6 oncoprotein recalls the *H. pylori* CagA-dependent and p53-mediated mechanism proposed for gastric carcinogenesis, thus reinforcing the hypothesis of a special PaCS role in it. Although HPV oncogenesis also depends on E7 oncoprotein interaction with the retinoblastoma tumor suppressor protein (Rb), it has been reported that E7 also binds to and activates ATPase subunit 4 of 26S proteasome, which may have a role in Rb degradation [57].

PaCSs have been observed in granulocytes from patients affected by Shwachman–Diamond disease [58] due to a mutation of the *SBDS* gene, known to take part in ribosome biogenesis and translation activation [59,60]. In this case, however, a large excess of pUbPs over proteasome content was detected in cell cytoplasm and even inside PaCSs, which were relatively poor in barrel-like particles, thus suggesting a relative insufficiency of proteasome degradative function in respect to an excessive accumulation of misfolded/denatured proteins caused by the *SBDS* gene mutation [61]. Indeed, decreased cell growth and increased apoptosis were found in such cells, whose impaired ribosome function might prevent the proteasome de novo biogenesis normally elicited by ubiquitinated protein deposition [62] and full development of proteasome particle-rich PaCSs.

From the above findings it appears that trophic factors and microbial oncoproteins may have an important role in the genesis of PaCSs and, possibly, of some PaCS-carrying neoplasms.

## 4. PaCS Induction in Cell Cultures under Trophic Factors/Interleukins Treatment

To obtain direct experimental evidence for a role of trophic factors and interleukins (ILs) in the genesis of PaCSs, we first investigated the process of human dendritic cell (DC) differentiation in vitro from their CD14^+^ peripheral blood precursors under treatment with GM-CSF plus IL-4 according to Sallusto and Lanzavecchia [63]. In this nonpathologic cell model, GM-CSF is known to directly activate the MEK-ERK signaling pathway [64], and IL-4 to display specific trophic activity on DC differentiation while inhibiting differentiation toward other APCs, such as macrophages [6,65,66,67]. During a 3–5 day treatment, we observed progressive development of PaCSs in DCs, from PaCS-free precursor cells up to their PaCS-filled derivatives showing full DC differentiation morphologically [26], although still “immature” in terms of antigen-presenting capacity [6,63]. Of note, the earliest, smallest PaCSs were found to arise inside ribosome-rich cytoplasm devoid of ER cisternae. No PaCSs were seen in parallel cultures of CD14^+^ monocytes left untreated or treated with GM-CSF alone, IL-4 alone, or GM-CSF plus INF-alpha, thus showing that PaCS-inducing capacity is restricted to the combination of trophic factors providing best DC differentiation and proliferation [26].

These findings directly establish a causative link between trophic factor/IL stimulation and PaCS development. A link is also supported by PaCS development in NK cells under treatment with IL-2 or IL-15 [21].

A PaCS development process, not unlike that seen in DCs and NK cells, was also found in megakaryoblasts from *ANKRD26*-mutated piastrinopenic patients under differentiation in vitro with thrombopoietin (TPO) plus IL-6 and IL-11, although no PaCS development was observed in equally treated megakaryoblasts from normal control subjects [68]. It has been shown that, in *ANKRD26*-mutated (but not in normal) megakaryoblasts, TPO/IL treatment elicits high, persistent MAPK-ERK expression, which, in turn, alters proplatelet formation, a necessary step in platelet release, strictly dependent, in normal megakaryoblasts, on a drop in MAPK-ERK pathway activity [69]. These findings, besides confirming a link between trophic factors/ILs and PaCS development, link it to the state of the MAPK-ERK pathway function.

These experimental findings fit with the in vivo occurrence of PaCSs in clinicopathological conditions or normal fetal tissue (see Section 3), where evidence has been obtained of proteasome, pUbPs, and Hsps overexpression in a background of enhanced cell proliferation and differentiation. Indeed, specific trophic factors/ILs are known to be involved, through pertinent signaling-pathway activation, in these cellular responses that require enhanced cell metabolism and protein renewal. This implies augmented production of misfolded proteins [16], possibly leading to PaCS development. This interpretation is also supported by available evidence of UPS-component overactivity, in addition to overexpression, either directly inside PaCSs [21] or in myeloid leukemia cells [28], where we found plenty of PaCSs [24].

## 5. PaCS Intracellular and Extracellular Fate

### 5.1. PaCS Intracellular Dissolution and Autophagy

**Figure 5:**
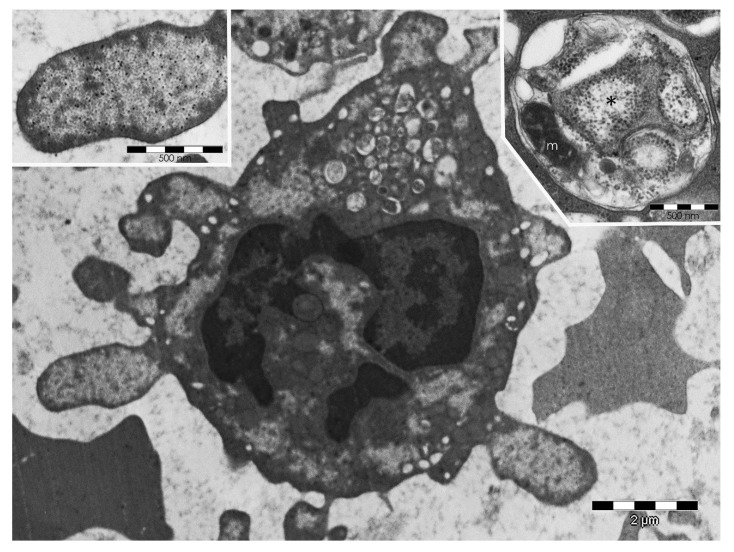
PaCS-filled blebs, ectosomes, and autophagic vesicles in myeloid-leukemia cells. Human bone-marrow biopsy showing a myeloid-leukemia cell with several cytoplasmic and bleb-filling PaCSs, one of which is enlarged in the top-left inset to show typical barrel-like particles and FK1 antibody pUbPs reactivity. In addition, note, in the bottom center of the cell, the detaching PaCS-bearing blebs forming extracellular ectosomes and, in the top-mid part of the cell, many autophagic vesicles. In the top-right inset, an autophagic vesicle from another myeloid-leukemia cell shows a distinctive double membrane enveloping a remnant of a small PaCS (asterisk), some ribosomes (free or attached to ER cisternae), and a mitochondrion (m).

Two kinds of PaCS changes were seen in vitro in fully differentiated DCs upon withdrawal of GM-CSF plus IL-4 incubation: (a) progressive loss of barrel-like particles up to a pattern of particle-empty PaCSs and to PaCS dissolution, and (b) autophagy of PaCSs, including residual particles and pUbPs, with final development of multiple membrane-enveloped cytoplasmic vesicles and cysts [26]. PaCS autophagy (Figure 5) was also a prominent finding in some human myeloid-leukemia cells [24], while PaCS-particle dissolution was also observed in the HL60 leukemia cell line treated with the E1 inhibitor Pyr-41 according to Xu and coworkers [28]. It seems that interruption of GM-CSF/IL-4 stimulation or of the excessive protein polyubiquitination inherent to leukemia [24], respectively, blocks the “compensatory” proteasome neogenesis [62] likely to aliment PaCS, thus leading to its emptying and dissolution, followed by autophagic removal of its remnants. This mechanism of PaCS dissolution seems interesting as, through it, PaCS components may contribute to other intracellular structures known to arise in variously stressed cells, such as, for instance, aggresomes [70,71], containing proteasome in addition to pUbPs and Hsps [72], or DC aggresome-like induced structures (DALIS), whose pUbPs may also have a role in antigen processing/transport before membrane presentation [26,73,74,75].

### 5.2. PaCS-Filled Cell Blebs

We observed PaCS-filled cytoplasmic blebs (Figure 5) under discharge (to form ectosomes) from a variety of neoplastic cells in vivo as well as in cultured cell lines and differentiating DCs or NK cells in vitro as well as from fetal cells in vivo [21,22,24,26]. This may be an easy way for a cell to eliminate excessive intracellular deposits of potentially toxic misfolded proteins, it might work as a discharge system of potential antigens to be taken up by immunocompetent cells, or it might even represent a sort of intercellular communication system acting through a “nonconventional secretory process” [24,76,77,78]. The high concentration of Hsp90 we found in PaCS-filled blebs and ectosomes [24] is of interest, as this Hsp has been shown to be secreted through a poorly definded “nonconventional” secretory process (to which PaCS-filled ectosomes might belong) by a number of neoplastic cells, of which it enhances the motility and invasive capacity [79].

## 6. PaCS versus Sequestosomes, Aggresomes, and Inclusion Bodies of Degenerative Diseases

**Figure 6:**
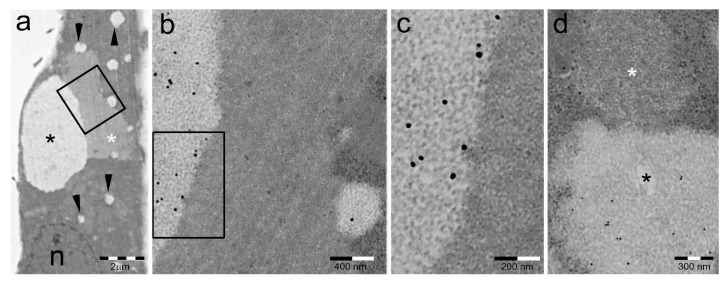
PaCSs and sequestosomes coexist in HeLa cells. (**a**) Ultrastructural identification of a sequestosome (white asterisk) adherent to a large PaCS (black asterisk) in the cytoplasm of a HeLa cell cultured under basal conditions. Note the presence of several small PaCSs (some with arrowheads). N, nucleus. The boxed area in (**a**) is enlarged in (**b**), and further in (**c**), to show PaCS′ distinctive barrel-like particles and FK1 antibody reactivity for pUbPs (see the immunogold particles on light gray areas) as opposed to the thin granulofibrillary structure and FK1 unreactivity of the sequestosome (no immunogold particles on dark gray area). (**d**) Another PaCS-adhering sequestosome (white asterisk) that is unreactive to proteasome immunogold, which labels the PaCS (black asterisk). Reproduced and adapted from Reference [81] under a Creative Commons Attribution (CC BY 3.0) License.

In addition to PaCS, HeLa cells also show at TEM investigation another kind of cytoplasmic structure characterized by a regular array of beaded granulofibrils, 5 to 8 nm thick, embedded in an amorphous, variably dense material (Figure 6). This “sequestosome” or “p62 body” [80] lacked any proteasome, FK1-antibody-positive pUbPs, or Hsp70 and Hsp90 reactivity, while reacting with p62/SQSTM1 protein antibodies and being susceptible to autophagic degradation [21]. Unlike PaCS, the sequestosome was easily preserved by conventional light- and confocal-microscopy procedures.

Despite their sharp ultrastructural and cytochemical differences (Table 1), HeLa-cell PaCSs and granulofibrillary sequestosomes were frequently found to be in direct continuity to each other while retaining ultrastructural individuality of their respective contents (Figure 6). Often, several focal deposits of granulofibrillary material were seen around the border of the same PaCS, a pattern suggesting special interaction between the two structures, such as multifocal deposition of a putative insoluble PaCS product escaping UPS degradation. Interestingly, thioflavin- and Congo red-positive aggregates of oligomeric E7 and/or E6 HPV proteins, known to be expressed by HeLa cells, have been obtained from HeLa and HPV-infected neoplastic cells [82,83].

Whether such aggregates of amyloid-like material have any relationship with the granulofibrillary sequestosome found at TEM remains to be investigated. However, oligomerization and aggregation of amyloidogenic proteins into thinly fibrillar precipitates, precursor of common amyloid fibrils, have been obtained in vitro [84,85], which closely resembled the thin fibrils of HeLa-cell sequestosomes [21,81]. In addition, it has been shown that, in amyloidogenic proteins containing proteasome-undegradable sequences like, for instance, expanded polyQ, proteasome limits its activity to the flanking soluble peptides, while leaving the undegradable inner sequences intact and free to undergo aggregation, precipitation, and fibrillation [86]. A similar sequence of events might account for the close association we found in HeLa cells between UPS-rich PaCS and sequestosome deposits.

Considerable cytochemical and ultrastructural similarities with HeLa-cell sequestosomes are shown by the hyaline bodies reported by Denk and coworkers [87] in hepatocellular carcinoma, characterized by a thinly fibrillar ultrastructure and heavy reactivity for p62/SQSTM1 protein and for Congo Red. Such hyaline bodies differ ultrastructurally and cytochemically from other structures of hepatocellular origin, such as the cytokeratin 8-reactive Mallory bodies, which seem more akin to aggresomes.

Perinuclear aggresomes, commonly arising in vitro in cells under various stressors [70] or in vivo in several degenerative diseases [88,89], are cytoplasmic bodies characterized by a variety of aggregated, denatured, mutated, and ubiquitinated proteins, together with the p62/SQSTM1 protein, with or without cytosolic or ER-resident chaperon molecules, as well as proteasomes [72,90,91]. They are also characterized by juxtanuclear topography due to special microtubule-dependent transport systems [70], a close relationship with lysosomes and autophagic vesicles [92,93], and by a rather polymorphous, compact-to-vesicular, partly disease-dependent ultrastructure. The juxtanuclear quality-control compartment (JUNQ) containing soluble, misfolded, and polyubiquitinated proteins, as well as proteasomes [94], has been interpreted as a reversible precursor form of aggresomes, to be distinguished from the “insoluble protein deposit” (IPOD), which is a yeast-sequestration compartment lacking association with proteasomes [95].

Despite their frequent sharing of proteasomes, ubiquitinated proteins, and chaperon molecules, aggresomes differ sharply from PaCSs for their polymorphous versus monomorphous particulate ultrastructure and for their easy preservation by aldehyde fixatives alone in the absence of osmium or additional fixatives [21]. In particular, in our TEM preparations we failed to detect, inside aggresomes, the regular network of proteasome-reactive barrel-like particles so characteristic of PaCSs. However, the possibility remains that PaCS-derived molecular components reach the aggresome when PaCS undergoes structural dissolution (see Section 5).

Aggresome formation has often been obtained in cells with proteasome inhibition or insufficiency [93,96,97]. In addition, a variety of cellular-inclusion bodies, interpreted as aggresomes [90,91,98,99], have been reported in several proteinopathies, including neurodegenerative diseases like Parkinson′s, Huntington′s, or Lafora disease, and cardiomyopathies, some of which showing evidence of proteasome insufficiency [96,100]. However, no PaCS-type structures have so far been found in cells and tissue from such conditions, despite PaCS detection in neuroblastic tumor cell lines [21]. On the contrary, UPS hyperfunction and/or hyperstimulation have been frequently documented in a variety of neoplastic diseases or fetal conditions [18,22,24,31,32,40] where we found much PaCS development, usually in the absence of aggresomes.

## 7. PaCS Biological and Pathological Role

The cytological and cytochemical investigations outlined above characterize PaCS as a UPS center formed by distinctive proteasome particles, to which pUbPs (likely of the K48-linked type) and several chaperon molecules (especially Hsp70 and Hsp90) are closely associated. PaCS-inducing experiments, using trophic factors on nonpathologic and pathologic cell lines, clearly documented its origin in connection with ER-free polyribosomes, i.e., at the site of cytosolic (nonsecretory) protein biosynthesis [26]. This finding, combined with early interaction of its proteasome/ubiquitin machinery with heat-shock proteins [101], points to PaCS as a focal expansion of the UPS involved in cytosolic-protein quality control, taking care especially of newly formed misfolded proteins [1,2,16,23].

Whenever tested, PaCS development was found to be associated with increased cell/tissue expression and/or UPS component activity, as well as with active cell proliferation and differentiation. In addition, inhibition of proteasome, ubiquitinating-enzyme, or related chaperon-molecule (Hsp90) activity has been tested with success as a potential therapeutic tool, and even introduced in clinical practice for some neoplastic diseases [28,29,30,38]. Therefore, in principle, a link between PaCS development and UPS overfunction seems likely, at least in neoplastic and fetal cells. However, it still remains an open point as to whether UPS overactivity is directly generated by the same factors (e.g., trophic factors, ILs, microbial oncogenic proteins) involved in generating neoplastic or fetal growth, or it is secondary to excessive production of misfolded, mutated, denatured pUbPs. In the latter case, the possibility of relative proteasome insufficiency (even if by itself quantitatively augmented) in respect to excessively increased protein-degradation demand should also be considered. A condition that is more akin to UPS stress rather than to simple hyperstimulation/hyperfunction, and with potential therapeutic implications.

Impaired function of the proteasome itself has also been considered as a potential contributor to insufficiency, including oxidative damage, especially to 19S regulatory particles [102], direct inhibition of proteasomal protease activities by misfolded prion protein oligomers [103], or indirect proteasome inhibition by amyloidogenic proteins aggregates [104,105], including “clogging” of the 26S proteasome particle channel. The latter was shown to be an unlikely event by Hipp and coworkers [106], who rather favored an impaired function of the cellular proteostasis network deputed to keep proteins in solution and prevent their aggregation.

Up to now, no PaCS-type structure has been found, in vivo or in vitro, in cells with actual evidence of proteasome insufficiency, either absolute or relative, and primary or secondary. Therefore, at present, PaCS remains linked essentially to conditions of increased cell proliferation/differentiation such as neoplasia and fetal development.

In conclusion, PaCS is a recently characterized UPS cytoplasmic structure, likely arising from its cytosolic protein-control compartment when, under increased functional demand (such as in preneoplastic, neoplastic, or fetal cells) due to increased proliferative and/or differentiation activity. PaCS detection in bioptic- or surgical-tissue samples should indicate ongoing UPS stress. However, the intimate mechanisms of PaCS′ in vivo formation, regression, or progression to pathologically relevant lesions largely remain to be clarified. In addition, PaCS′ role in processing endogenous, cytosolic proteins of potentially antigenic power for subsequent presentation in an MHC background remains to be specifically investigated.

## Figures and Tables

**Table 1:** Comparison of PaCS and sequestosome features.

	PaCS	Sequestosome
**Ultrastructure** *	Collection of barrel-like particles	Granulofibrillar arrays
**Content**		
(a) Proteasome	Yes	No
(b) Polyubiquitinated proteins	Yes (likely K48-linked)	No
(c) Hsp 70 and 90	Yes	No
(d) P62/SQSTM1	No	Yes
**Degradation by autophagy**	Possible	Possible
**Entering cell blebs and ectosomes**	Frequent	Not found
**Associated pathology**	Clear cell neoplasms	Hepatocellular cancer

* See also Figure 6.

## Abbreviations

APC	antigen-presenting cell
DALIS	DC aggresome-like induced structures
DC	dendritic cell
EGFR	EGF receptor
ER	endoplasmic reticulum
ERAD	endoplasmic reticulum-associated degradative
HIF	hypoxia-inducible factor
Hsp	heat-shock protein
IPOD	insoluble protein deposit
JUNQ	juxtanuclear quality-control compartment
NK	natural killer
PaCS	particulate cytoplasmic structure
PSCN	pancreatic serous cystic neoplasm
pUbPs	polyubiquitinated proteins
Rb	retinoblastoma tumor-suppressor protein
TAP	transporter for antigen processing
TEM	transmission electron microscopy
TPO	thrombopoietin
UPS	ubiquitin–proteasome system
VHL	Von Hippel–Lindau
